# Bacterial heteroresistance mechanisms, dynamics, and emerging diagnostic approaches

**DOI:** 10.1042/BSR20260035

**Published:** 2026-07-03

**Authors:** Sophie Brameyer

**Affiliations:** Faculty of Biology, Microbiology, Ludwig-Maximilians-University München, Großhaderner Str. 2-4, 82152 Martinsried, Germany

**Keywords:** antibiotic resistance, antibiotics, Antimicrobial susceptibility testing, Heteroresistance, Phenotypic resistance

## Abstract

Antibiotic heteroresistance is a form of within-isolate susceptibility heterogeneity in which an apparently susceptible bacterial population contains rare subpopulations capable of growth at substantially higher antibiotic concentrations. It is commonly defined as resistant minorities occurring at frequencies ≥10^−7^ and growing at concentrations at least eight-fold above those that inhibit the dominant population, as demonstrated by population analysis profiling or related assays. This phenomenon has been described in diverse Gram-negative and Gram-positive pathogens and across multiple drug classes and is frequently characterized by instability, with resistant subpopulations expanding under treatment and contracting once drug pressure is relieved. This dynamic behavior contributes to systematic under-detection by routine antimicrobial susceptibility testing, which is optimized for population-average endpoints and may miss clinically relevant resistant tails, helping explain treatment failure despite ‘susceptible’ results. Mechanistically, heteroresistance spans a continuum from genotypic processes, including gene amplification, plasmid copy-number variation, transposition, and point mutations, to phenotypic mechanisms driven by reversible regulatory and physiological states such as transcriptional reprogramming, stochastic expression variability, and intergenerational phenotypic memory. These mechanisms can coexist and shift under antibiotic selection, supporting the view that heteroresistance may act as an evolutionary intermediate linking susceptibility and stable resistance. Recent advances in single-cell phenotyping, copy-number-aware genomics, transcriptomics, pharmacodynamic and population modeling, and machine-learning-assisted diagnostics offer new opportunities to detect and interpret susceptibility distributions. Integrating these approaches into clinical microbiology and stewardship will be essential to improve risk stratification, guide therapy, and predict resistance evolution.

## Introduction

Many bacterial species and antibiotic classes exhibit heteroresistance, a phenomenon in which a susceptible bacterial isolate harbors a resistant minority subpopulation that can grow in the presence of an antibiotic and cause treatment failure. The resistant phenotype is often unstable, and without antibiotic selection, it reverts to susceptibility. These resistant minority subpopulations are capable of growth at substantially higher antibiotic concentrations than the bulk population. Operational definitions commonly require resistant subpopulations at frequencies at or above 10^−7^, which grow at antibiotic concentrations at least eight-fold higher than those that inhibit the dominant population, as quantified by population analysis profiling (PAP) or related assays [1,2]. Heteroresistance has been documented across Gram-negative and Gram-positive pathogens and across diverse antibiotic classes and is frequently unstable and reversible, allowing resistant minorities to expand during therapy yet regress when drug pressure is removed [1,3,4]. Clinically, heteroresistance can undermine routine antimicrobial susceptibility testing (AST) and contribute to treatment failure despite a ‘susceptible’ categorization, motivating improved conceptual frameworks and diagnostics [1,4,5].

Mechanistically, heteroresistance spans a continuum from genotypic heteroresistance, where minority resistant cells harbor detectable genetic alterations, to phenotypic heteroresistance [2,6–8]. Recent work has sharpened this dichotomy while also emphasizing coexistence: gene dosage-dependent heteroresistance is prominent in many Gram-negative species, whereas, for example, in *Staphylococcus aureus*, heteroresistance can be driven by diverse point mutations in core genes. In parallel, lineage-resolved single-cell studies show that phenotypic resistance can be selectively inherited across generations even in genetically identical populations [9,10], showing that heteroresistance mechanisms are likely more intricate than genetic predisposition alone [8]. Across multiple drug–pathogen pairs, resistant minorities are not merely laboratory artifacts: animal models and clinical case series support that small resistant fractions can increase failure risk and can seed evolution to stable resistance under selection [1,2,11,12]. This was also demonstrated for carbapenem-resistant Enterobacterales, where heteroresistance to β-lactams can emerge gradually over years of antibiotic use and eventually lead to stable, homogeneous resistance [13]. Conceptually, this implies that heteroresistance can be a transitional stage on the path from susceptibility to full resistance, under sustained selection pressure, as resistant subpopulations are progressively enriched until resistance becomes stable and widespread across the population.

Broad surveys and reviews indicate that heteroresistance is common across diverse pathogens and antibiotic classes [1,2,4]. However, the ‘true prevalence’ remains uncertain because studies differ in heteroresistance definitions, screening techniques, and confirmatory methods, and because many reports draw on biased collections enriched for multidrug-resistant isolates [5]. Even among studies using PAP and referencing the Andersson et al. framework, reported resistant-subpopulation frequencies often fall in the 10^−7^ to 10^−5^ range, meaning that modest differences in sampling depth or workflow can flip a classification [1–4]. Consequently, currently used heteroresistance thresholds should be viewed as operational definitions that facilitate comparison between studies rather than biologically absolute boundaries.

The present review synthesizes current concepts in antibiotic heteroresistance in bacteria, including definitions, evolutionary and mechanistic frameworks, epidemiology, and clinical implications. Key topics covered include the dynamics of genotypic and phenotypic heteroresistance, detection challenges, and emerging diagnostic strategies, with emphasis on population-resolved phenotyping, omics integration, pharmacodynamic modeling, and AI-assisted clinical triage.

## Antibiotic heteroresistance

Antibiotic treatment confronts not only species-level resistance but also substantial heterogeneity within clonal bacterial populations. Clinical microbiology often summarizes isolates using a single minimum inhibitory concentration (MIC), yet bacterial populations can exhibit heterogeneous survival and growth strategies that are not captured by a single-point metric [1,6]. Heteroresistance is distinct in that minority subpopulations are not merely surviving transient exposure (as in tolerance or persistence) but can actively grow at antibiotic concentrations substantially higher than those inhibiting the dominant population [1]. Because resistant minorities can occur at very low frequencies, standard AST workflows may fail to detect them, potentially resulting in misleading susceptibility classifications [3,4]. This discordance between routine categorical AST and the true distribution of susceptibilities is increasingly recognized as a systematic phenomenon rather than isolated ‘odd’ results and has been explicitly highlighted as a cause of discrepant AST outcomes in clinical practice [5].

Experimental studies, modeling, and clinical associations link heteroresistance to treatment failure in some settings, particularly when antibiotic exposure selects for expansion of the minority resistant fraction [1,4,14]. An important conceptual shift is the recognition that treatment failure in this context is inherently probabilistic, since the likelihood of failure depends on factors such as the size of the resistant fraction, drug exposure at the infection site, bacterial density, and host conditions when resistant minorities occur at frequencies ranging from approximately 10^−2^ and 10^−6^ [1,15]. However, robust prospective human clinical studies directly establishing causality between heteroresistance and therapeutic failure remain comparatively limited, and clinical outcome data are still evolving.

Two developments have sharpened the modern heteroresistance discourse. First, mechanistic studies in Gram-negative pathogens show that heteroresistance is frequently driven by gene dosage changes, like tandem amplification, plasmid copy number increases, and transposition to higher-copy replicons, producing unstable resistance that can expand quickly under selection and regress without it [2,3,7]. Second, research focused predominantly on Gram-positive bacteria, especially in *S. aureus*, demonstrates that heteroresistance can also arise from diverse point mutations in core genes, broadening the mechanistic repertoire beyond copy number variation [9]. Parallel single-cell approaches indicate that even in the absence of stable genetic changes, bacteria can express heritable phenotypic resistance over multiple generations, implying that phenotypic heteroresistance can be partially inherited and selected [10].

## Definitions and conceptual boundaries

A widely used operational definition of heteroresistance requires that an isolate contains a resistant subpopulation at a frequency of at least ≥10^−7^ that can grow at antibiotic concentrations at least eight-fold higher than the concentration inhibiting the dominant susceptible population [1,2,16]. This definition is explicitly population-based and is most naturally evaluated by PAP, which quantifies colony-forming units across an antibiotic concentration gradient [1,2]. The eight-fold separation is intended to exceed typical assay variability around breakpoints, and the frequency threshold reflects practical detectability in plating-based assays. However, clinically relevant resistant fractions often lie between approximately 10^−2^ and 10^−6^, where the probability of treatment failure becomes a function of resistant fraction size, total bacterial burden, site-specific drug exposure, and host factors, rather than a simple binary susceptible/resistant classification [1,15].

**Figure 1:**
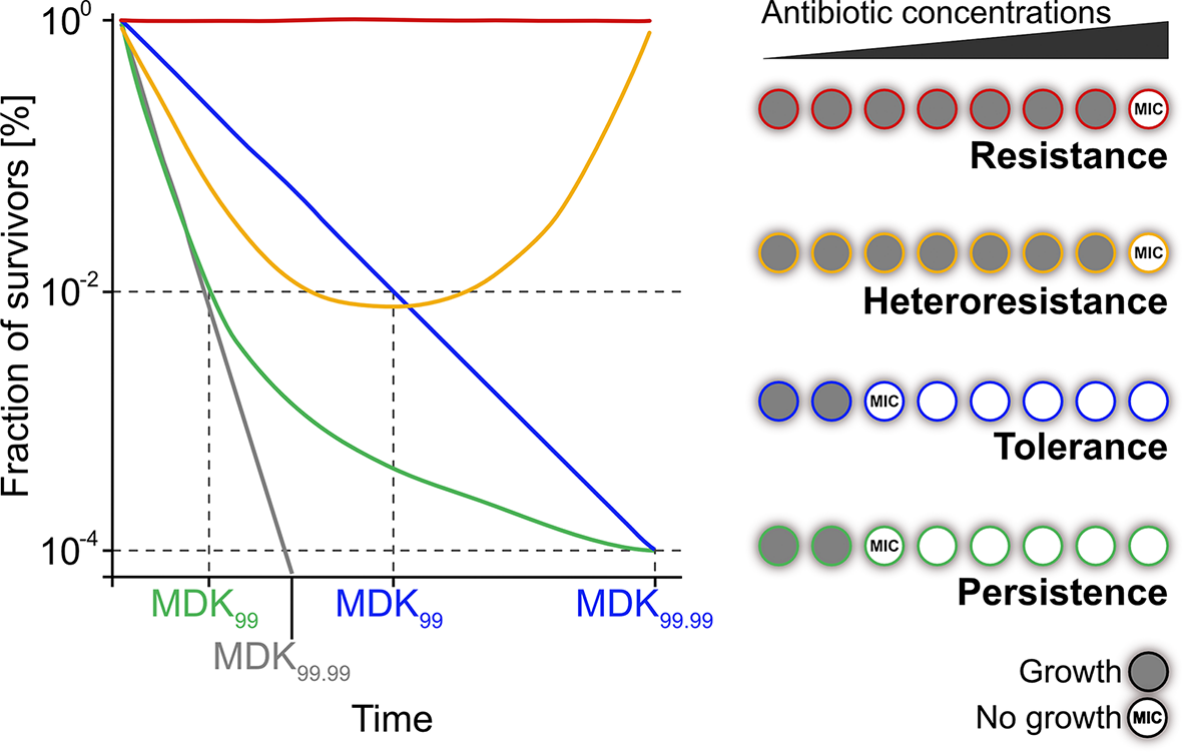
Schematic comparison of resistance, heteroresistance, tolerance, and persistence **Left:** Time-kill dynamics showing the fraction of survivors in bacterial populations exposed to an antibiotic. Resistant populations (red) show little or no loss of viability. Heteroresistant populations (orange) exhibit biphasic killing due to a resistant subpopulation, with initial killing followed by regrowth of resistant cells during continued antibiotic exposure; the apparent MIC is similar to that of a fully resistant population. Tolerant populations (blue) have an MIC comparable to susceptible populations (gray) but are killed more slowly, resulting in an increased minimum duration for killing 99% of the population (MDK_99_). Persistent populations (green) also have an MIC and MDK_99_ similar to susceptible populations, but a small subpopulation survives for prolonged periods, producing a pronounced tail and a markedly increased MDK_99.99_ (minimum duration for killing 99.99% of the population). **Right:** Schematic broth microdilution across increasing antibiotic concentrations (left to right). Resistance and heteroresistance shift the MIC to higher concentrations, whereas tolerance and persistence leave the MIC largely unchanged but alter killing kinetics. Dark-grey wells indicate growth; white wells indicate no growth (inhibition) at or above the MIC.

Heteroresistance is often confused with several adjacent concepts. Homogeneous resistance refers to a bulk population that meets resistance criteria, whereas heteroresistance involves a resistant minority embedded within a susceptible majority [1]. Tolerance denotes slowed killing without an MIC shift, while persistence refers to rare phenotypic states that survive antibiotics, typically without robust growth under exposure [6]. In contrast, heteroresistant minorities can grow at antibiotic concentrations that inhibit the main population, making the phenotype potentially more directly linked to regrowth during therapy (Figure 1) [1,6]. Thus, the key operational boundary is proliferative capacity: heteroresistant subpopulations form growing colonies or expanding populations at antibiotic concentrations that suppress the dominant susceptible population, whereas tolerant or persistent cells are primarily detected by delayed killing or prolonged survival without sustained net growth.

## A mechanistic framework for genotypic and phenotypic heteroresistance

**Figure 2:**
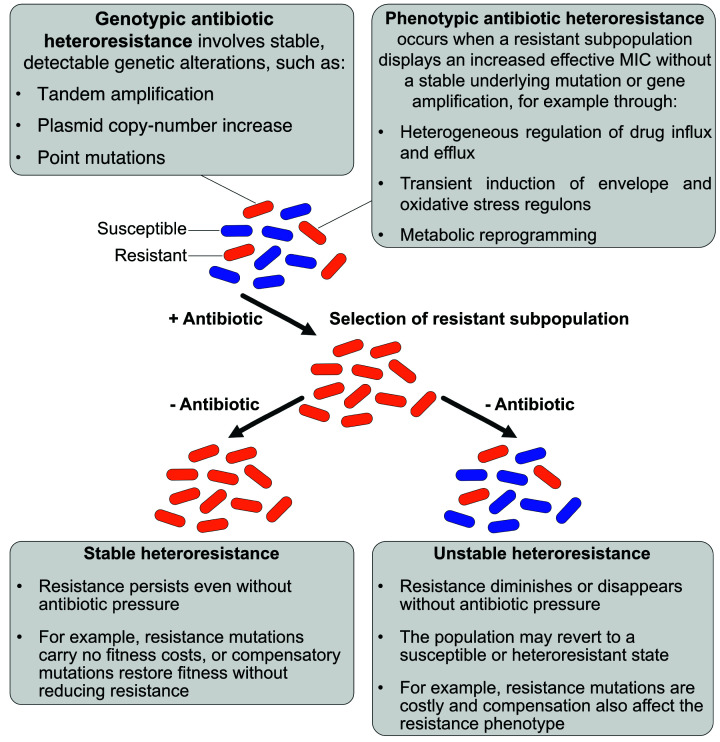
Genotypic and phenotypic heteroresistance and their stability Schematic overview of mechanisms underlying heteroresistance and their consequences for population dynamics. Genotypic heteroresistance is driven by stable, detectable genetic alterations, such as tandem gene amplification, increased plasmid copy number, or point mutations. In contrast, phenotypic heteroresistance arises from reversible, non-genetic mechanisms that result in a transient increase in the effective MIC, for example, through heterogeneous regulation of drug influx and efflux, transient activation of envelope or oxidative stress responses, or metabolic reprogramming. Upon antibiotic exposure, resistant subpopulations are selected and can dominate the population. After removal of antibiotic pressure, two outcomes are possible. In stable heteroresistance, resistance persists in the absence of an antibiotic, typically due to low fitness costs or compensatory mutations that maintain resistance. In unstable heteroresistance, resistance diminishes or disappears when antibiotic pressure is removed, leading to reversion to a heteroresistant or susceptible population. Blue cells indicate susceptible bacteria, and orange cells indicate resistant bacteria.

Heteroresistance is increasingly recognized as a distributional phenotype that can arise through both genetic and non-genetic mechanisms [1,6,8–10]. While operational definitions focus on the presence of resistant minority subpopulations, growing evidence indicates that these populations may emerge through a continuum of underlying mechanisms ranging from stable genetic alterations to reversible physiological states [1,2,6,8]. Consequently, heteroresistance is best viewed not as a single mechanism but as a population-level phenotype that can be generated through distinct yet sometimes overlapping biological processes [1,6,10]. A practical mechanistic dichotomy distinguishes genotypic from phenotypic heteroresistance (Figure 2). Genotypic heteroresistance occurs when the resistant subpopulation carries detectable genetic changes such as tandem amplification, plasmid copy-number increase, or point mutations. Resistance is heritable as long as the genetic state persists; in unstable mechanisms, reversion can restore susceptibility [2,7,9]. Phenotypic heteroresistance occurs when the resistant subpopulation exhibits higher effective MIC without a stable underlying mutation or amplification; differences can be generated by heterogeneous regulation of drug influx and efflux (including variable porin abundance and efflux pump activity), transient induction of envelope and oxidative stress regulons, and metabolic reprogramming that slows growth or alters target availability (Figure 2). Phenotypes may be partially inherited over a few generations and then revert in drug-free conditions [6,8,10]. Recent synthesis of phenotypic heterogeneity emphasizes that such non-genetic diversity is not incidental noise but a selected population-level trait that enables survival under fluctuating antibiotic stress, particularly through persistence- and heteroresistance-associated physiological states [17]. These forms can coexist within a single isolate, and the balance can shift during antibiotic selection and laboratory passaging.

## Genotypic heteroresistance mechanisms

First, one major mechanism underlying heteroresistance is gene dosage variation caused by tandem amplification of resistance loci. This process transiently increases gene copy number and expression in a subset of cells, resulting in elevated effective MICs within the population. Because amplified genomic regions frequently carry a metabolic burden, these states are typically unstable and can rapidly collapse through recombination or copy-number reduction once antibiotic pressure is relieved (Figure 2) [2,3]. Choby and colleagues reported that gene copy-number flexibility can facilitate heteroresistance under increasing antibiotic pressure and undermine the β-lactam development/testing pipeline. They showed that cefiderocol heteroresistance in clinical Enterobacter isolates can be driven by β-lactamase gene amplification, producing a continuum of resistant subpopulations observed even among isolates collected before cefiderocol’s clinical introduction. Stepwise increases in copy number of an extended-spectrum-β-lactamase gene with intrinsically poor activity against cefiderocol shift susceptibility upward in progressively smaller fractions of cells. The authors further showed that amplification-mediated phenotypic flexibility can support adaptation to β-lactam/β-lactamase-inhibitor combinations (e.g., cefiderocol/avibactam) without requiring stable evolutionary change [18].

Second, another mechanism for genotypic heteroresistance is an increase in plasmid copy number, which transiently elevates the dosage of resistance determinants in a subset of cells. This stochastic variation can generate small subpopulations capable of growth at antibiotic concentrations that inhibit the dominant population. A clinical example is *Klebsiella pneumoniae* heteroresistant to piperacillin/tazobactam, mediated by increased copy number of multiple plasmid-borne β-lactamase genes (e.g., bla variants) [19]. These observations are consistent with a dosage-threshold framework, where only cells that transiently exceed a critical expression level can survive or proliferate during antibiotic exposure. Plasmid copy-number variation has also been implicated in heteroresistance to newer agents such as cefiderocol. In pandrug-resistant, hypervirulent *K. pneumoniae* ST11, cefiderocol heteroresistance (and shifts toward resistance) was associated with variable copy numbers of *bla_SHV-12_* carried on IncR/IncFII plasmids. Thereby, higher copy number correlated with higher cefiderocol MICs, consistent with a gene-dosage continuum in which increasingly resistant subpopulations occur at progressively lower frequencies [20].

More broadly, experimental studies indicate that intrinsic variation in plasmid abundance among individual cells can produce heterogeneous resistance gene expression, thereby increasing the probability that rare variants persist during antibiotic treatment. This effect provides a general mechanistic basis by which plasmid copy-number heterogeneity can manifest as heteroresistance-like resistant tails in population assays [21]. In 2024, Nicoloff and colleagues investigated a multidrug-resistant *K. pneumoniae* isolate and identified three main gene-dosage-based mechanisms driving heteroresistance, including tandem amplification, increased plasmid copy number, and transposition of resistance determinants onto additional plasmids. They showed that these mechanisms can co-occur within the same genetic background, impose measurable fitness costs, and revert quickly once antibiotic selection is removed. Using a mouse gut colonization model, they further provided evidence that elevated resistance gene dosage and the resulting heteroresistance can promote antibiotic treatment failure, consistent with the clinical concern that initially rare resistant subpopulations can rapidly expand during therapy [7].

Thirdly, heteroresistance can be generated via point mutations, and one common route is target modification. In *Mycobacterium tuberculosis*, rifampicin heteroresistance is a canonical example in which low-frequency variants of *rpoB* coexist with wild-type bacilli and can be selectively enriched during rifampicin exposure, linking apparent susceptibility to on-therapy emergence of overt resistance [22,23]. A similar logic applies to fluoroquinolones, where heteroresistant subpopulations can reflect minority variants in quinolone target genes, such as *gyrA*/*gyrB* in *M. tuberculosis* or often *gyrA*/*parC* in other bacteria [24,25]. A second, highly prevalent route is regulatory rewiring, in which point mutations (or small disruptive variants) in global regulators and two-component systems shift envelope composition, stress responses, and/or efflux capacity, yielding a resistant subpopulation that can expand during treatment and contract when selection is removed. In *K. pneumoniae*, colistin heteroresistance and resistance are repeatedly linked to lesions in *mgrB*, encoding a negative regulator of PhoPQ, and mutations affecting PhoPQ/PmrAB-connected signaling, which drive lipid A remodeling and reduce polymyxin binding [26–28]. Extending this point-mutation framework to Gram-positive pathogens, studies of clinical *S. aureus* isolates have found heteroresistance to multiple antibiotics to be common, yet often not attributable to amplification of canonical resistance genes. Instead, resistant subpopulations were linked to diverse point mutations in core chromosomal genes, implying a broad mutational target space and supporting the idea that heteroresistance can persist as a stable, low-frequency genetic minority maintained by fitness trade-offs in antibiotic-free conditions but poised to expand under treatment [9]. Finally, point mutations often contribute to heteroresistance as early steps along stepwise, polygenic adaptive paths. Here, heteroresistance reflects an evolutionary intermediate in which multiple loci can be sampled in parallel within an isogenic population, and sublineages with partial resistance phenotypes persist at low frequency until antibiotic pressure favors their expansion and additional mutations accumulate. The best-studied illustration is the glycopeptide heteroresistance in heterogeneous vancomycin-intermediate *S. aureus* (hVISA) and its progression to vancomycin-intermediate *S. aureus* (VISA). Recurrent mutations in regulatory systems controlling cell-wall metabolism and envelope stress responses, particularly in *walKR*, *vraSR*, and *graRS*, as well as in global transcriptional machinery such as *rpoB*, have been repeatedly linked to intermediate and heterogeneous vancomycin phenotypes [29–31]. This polygenic architecture helps to explain why heteroresistance is often dynamic and context-dependent, with detection and apparent stability varying with factors such as medium composition, inoculum, and prior drug exposure history, and why it may fluctuate as resistant subpopulations expand under antibiotic selection and contract once selection is removed [3,32,33]. Representative examples of genotypic heteroresistance mechanisms are summarized in Table 1.

**Table 1:** Representative examples of genotypic and phenotypic heteroresistance mechanisms

Pathogen	Antibiotic	Mechanism	Classification
*Klebsiella pneumoniae*	Colistin	*mgrB*/PhoPQ alterations	Genotypic
*Klebsiella pneumoniae*	Cefiderocol	*blaSHV-12* amplification	Genotypic
*Klebsiella pneumoniae*	Piperacillin/tazobactam	Increased β-lactamase plasmid copy number	Genotypic
*Staphylococcus aureus*	Vancomycin	*walKR*, *vraSR*, *graRS* mutations	Genotypic
*Mycobacterium tuberculosis*	Rifampicin	Minority *rpoB* variants	Genotypic
*Escherichia coli*	Imipenem	Rapid transcriptional adaptation	Phenotypic
*Pseudomonas aeruginosa*	Polymyxins	Adaptive lipid A modification responses	Phenotypic
*Acinetobacter baumannii*	Polymyxin B	Exposure-induced adaptive resistance	Phenotypic
*Staphylococcus aureus*	Oxacillin/Methicillin	Heterogeneous *mecA* expression	Phenotypic

Representative pathogen–antibiotic pairs illustrating the mechanistic diversity of bacterial heteroresistance. Genotypic heteroresistance is associated with detectable genetic alterations, including gene amplification, plasmid copy-number variation, transposition events, and point mutations. Phenotypic heteroresistance arises from reversible physiological or regulatory states in genetically identical populations and may involve adaptive transcriptional responses, heterogeneous expression of resistance determinants, stress-response activation, or altered antibiotic accumulation. The examples shown are representative rather than exhaustive and correspond to mechanisms discussed throughout the review. Classification follows the mechanistic framework outlined in Figure 2.

Amplification-based heteroresistance commonly imposes a fitness cost in antibiotic-free environments because maintaining extra copies of resistance genes increases the replicative and transcriptional burden and can reduce competitive performance relative to lower-copy counterparts. In 2024, Pal and Andersson demonstrated that compensatory evolution can mitigate the fitness costs of resistance-gene amplification, thereby facilitating the transition from copy-number-mediated heteroresistance to stable resistance [34]. Gene amplification represents a readily accessible and frequently reversible adaptive strategy that broadens phenotypic diversity under antibiotic stress, increasing the likelihood that resistant lineages are maintained during treatment. Continued selection can then favor compensatory changes that reduce the burden of high resistance and/or stabilize resistance through additional mutations [35,36]. Consistent with heteroresistance as an evolutionary intermediate, clinical evidence in *Escherichia coli* and related Enterobacterales indicates that β-lactam heteroresistance can emerge before stable resistance becomes prevalent and may be gradually supplanted by fixed resistance over time [13]. Together, these findings support an evolutionary narrative in which heteroresistance enables survival and amplification of resistant minorities during therapy, while compensatory and stabilizing genetic changes progressively convert a costly, reversible dosage phenotype into stable, high-level resistance that can fix in the population.

## Phenotypic heteroresistance and non-genetic heterogeneity

Phenotypic heteroresistance describes situations in which a genetically uniform bacterial population contains a minority subpopulation with substantially higher effective antibiotic resistance that arises from reversible physiological states rather than stable genetic changes (Figure 2). In practice, this can yield an apparently ‘susceptible’ bulk MIC while still permitting survival and regrowth under therapy, because the resistant tail can be rapidly enriched during antibiotic exposure and then contract once selection is removed. Thus, phenotypic heteroresistance reflects a transient shift in cellular physiology that alters intracellular drug exposure or drug-target engagement, rather than the acquisition of canonical resistance determinants [1,3,16,37]. Representative phenotypic heteroresistance mechanisms are shown in Table 1.

Mechanistically, phenotypic heteroresistance can be generated by heterogeneous regulation of drug influx and efflux (including variable porin abundance and efflux pump activity), transient induction of envelope and oxidative stress regulons, and metabolic reprogramming that slows growth or alters target availability, thereby reducing antibiotic lethality at a given extracellular concentration. These processes are shaped by regulatory noise, environmental sensing, and stress history, linking phenotypic heteroresistance to broader frameworks of bacterial individuality and non-genetic heterogeneity that also encompass tolerance and persistence [6]. Consequently, phenotypic heteroresistance is often highly conditional, as the size of the resistant subpopulation depends strongly on growth conditions, medium composition, inoculum size, growth phase, and prior exposure history [2–4,16,32,38]. Additional mechanistic examples illustrate how noncanonical cellular functions can shape heterogeneity. Heterogeneity in responses to ribosome-targeting antibiotics has been linked to bacterial RNA repair activity, implying that state-dependent repair capacity can create cell-to-cell variability in antibiotic outcomes and broaden the mechanistic landscape for phenotypic heteroresistance-like distributions [39].

Phenotypic heteroresistance can span pathogens and drug classes. In *S. aureus*, heterogeneous methicillin and oxacillin resistance is a classic population-level example in which only a minority of cells expresses high-level β-lactam resistance at any given time, reflected by heterogeneous expression of *mecA* and modulation of the PBP2a-mediated resistance program. Under β-lactam exposure, these resistant minorities can be selectively amplified even when routine susceptibility testing underestimates resistance expression [40,41]. In Gram-negative bacteria, adaptive resistance to polymyxins provides an instructive analogue of phenotypic heteroresistance. For example, *Pseudomonas aeruginosa* can rapidly transition into a less susceptible state through inducible regulatory responses, including two-component systems that drive lipid A modification pathways, with resistance reverting after drug withdrawal [42]. Similar hetero- and adaptive resistance phenotypes to polymyxin B have been documented in carbapenem-resistant *Acinetobacter baumannii*, where resistant subpopulations and exposure-induced increases in reduced susceptibility are detectable *in vitro*, underscoring the role of non-genetic heterogeneity and exposure history in shaping susceptibility [43,44].

Beyond polymyxins, rapid, non-genetic heteroresistance behavior has been described for carbapenems in *E. coli*, where imipenem-adaptive subpopulations emerge on short timescales through dynamic transcriptional regulation rather than acquisition of carbapenemases or target mutations. These observations highlight how residual antibiotic exposure can preferentially enrich pre-existing adaptive minorities and complicate the interpretation of susceptibility results derived from bulk population measurements [8]. Population-resolved and single-cell measurements further support plausible physiological bases for phenotypic heteroresistance, as heterogeneous antimicrobial accumulation driven by variable efflux activity can generate minority subpopulations that limit intracellular drug exposure and evade lethal damage even in clonal cultures, as shown for the antimicrobial peptide tachyplesin in *E. coli* and *P. aeruginosa* [45].

Importantly, phenotypic heteroresistance need not be purely momentary noise. Single-cell lineage tracking reveals that susceptibility states can exhibit partial inheritance, such that resistant lineages persist across generations long enough to be selectively enriched during antibiotic exposure even without genetic change. This ‘phenotypic memory’ provides a mechanistic bridge between transient physiological states and population-level heteroresistance dynamics, strengthening the plausibility of phenotypic heteroresistance as a stable population property with history dependence [10].

Placing phenotypic heteroresistance within the broader landscape of non-genetic heterogeneity also clarifies its relationship to tolerance and persistence. All three reflect within-population variability, but they are operationally distinguished by experimental readouts: heteroresistance is typically defined by a resistant subpopulation with a substantially elevated apparent MIC relative to the main population, whereas tolerance and persistence are often inferred from killing kinetics without a corresponding MIC shift (Figure 1). Accordingly, mechanistic overlap should not be interpreted as equivalence, as stress responses or metabolic slowing may contribute to all three phenotypes, but only heteroresistance is defined by a minority fraction with an increased growth-permissive susceptibility threshold. Nonetheless, these phenotypes overlap mechanistically, for example, through stress responses, metabolic slowing, and heterogeneous gene expression. Thereby, MIC-centered diagnostics can overlook minority behaviors that are clinically relevant under fluctuating or prolonged drug exposure. Collectively, this framing supports treating phenotypic heteroresistance not as a laboratory curiosity but as a clinically meaningful component of bacterial survival strategies that can contribute to discordance between ‘susceptible’ test results and suboptimal therapeutic outcomes [1,3,16,37].

## Interactions between phenotypic and genotypic heteroresistance and implications for therapy and stewardship

Antibiotic exposure enriches resistant minorities generated by genotypic or phenotypic heterogeneity, and resistance either reverts when costly and unstable or stabilizes through compensatory evolution or additional mutations [3,46]. Gene amplification and copy-number variation are now recognized as central, quantitatively important contributors to heteroresistance across multiple pathogens, providing a reversible but selectable mechanism that bridges phenotypic and genotypic heteroresistance [2,34]. Recent work further shows that fitness costs of amplified resistance can be mitigated by compensatory changes, facilitating persistence and eventual stabilization of resistance under continued exposure [9]. Thereby, phenotypic heteroresistance can act as a short-term survival bridge under sudden exposure, while genotypic heteroresistance provides a longer-lived but still reversible solution, increasing the probability of transition to stable resistance. Clinically, heteroresistance is most concerning when antibiotic exposure is heterogeneous or suboptimal, bacterial burden is high, and pathogen-drug pairs with documented heteroresistance prevalence are involved [1,4,37]. Importantly, the clinical relevance of heteroresistant minorities depends not only on intrinsic bacterial mechanisms but also on pharmacokinetic and pharmacodynamic factors such as tissue-specific drug penetration, fluctuating antibiotic exposure, spatial antibiotic gradients, bacterial density, and host factors [14,15]. These dynamics argue for stewardship strategies that minimize selective underexposure, optimize dosing, and reserve combination therapy for contexts where heteroresistance minorities are likely to expand, while research priorities include standardized definitions, scalable high-throughput phenotyping, copy-number-aware genomics, and externally validated machine-learning-based decision-support tools [16,47].

## Methods, limitations, and emerging approaches in detecting heteroresistance

Once regarded as a laboratory curiosity, heteroresistance is now recognized as a clinically relevant explanation for discordance between genotypic resistance prediction and phenotypic AST, as well as for unexpected treatment failure under apparently appropriate therapy [4,48]. A central difficulty in detection is that heteroresistant subpopulations are often rare, unstable, and highly sensitive to growth conditions, antibiotic exposure history, and sampling depth, making them poorly aligned with routine diagnostics designed to measure dominant population behavior at fixed endpoints [2,3]. Routine AST methods, including disk diffusion, broth microdilution, gradient diffusion, and automated systems, frequently underestimate or entirely miss heteroresistance [3,4]. Resistant minorities may fall below sampling thresholds, be counterselected during drug-free subcultures, or fail to expand within standardized incubation times [3,48]. As a result, heteroresistance is increasingly framed as a category mismatch: conventional AST assigns categorical interpretations based on average or dominant-population responses, whereas heteroresistance is fundamentally a property of the distribution tail [5]. In this context, negative routine AST results do not exclude the presence of clinically relevant resistant minorities [3,4].

Among available assays, PAP (often reported as PAP-AUC, population analysis profile-area under the curve) remains the most widely accepted reference and confirmatory method for detecting heteroresistance because it directly quantifies growth of subpopulations across antibiotic gradients [1,4]. PAP provides a population-resolved view of susceptibility and is formally standardized for certain phenotypes, most notably for hVISA [49,50]. However, PAP is labor-intensive, slow, and difficult to scale for routine clinical use [4]. Moreover, even PAP results can be ambiguous, as heteroresistance-like patterns may arise from inoculum effects, transient adaptive states, or methodological variability [4,16,50]. These limitations highlight that the ‘ground truth’ of heteroresistance is partly dependent on pre-analytical handling, culture conditions, and interpretive criteria [2,3]. Intermediate screening approaches, such as identifying colonies within inhibition zones on gradient diffusion tests, using screening agar plates at critical antibiotic concentrations, or relying on automated system flags, can improve sensitivity in selected settings but exhibit variable specificity. Comparative evaluations show that different screening assays detect overlapping but non-identical heteroresistant subpopulations, reinforcing that the absence of detection in routine workflows does not rule out heteroresistance potential [4,50]. Epidemiological studies further highlight method dependence, with reported hVISA/vancomycin heteroresistance prevalence varying widely across regions [51–53]. Meta-analyses confirm that heteroresistance is globally distributed and that prevalence estimates are strongly shaped by detection methodology and isolate selection [54].

Sampling dependence and inoculum effects represent additional diagnostic blind spots. When resistant minorities are present at very low frequency, their detection depends on whether sufficient cells are sampled and on how antibiotic exposure scales with bacterial density [32,38]. Experimental and clinical microbiology studies demonstrate that modest differences in inoculum size can substantially shift measured MICs and alter categorical interpretations [32,38]. In heteroresistance, these factors are not nuisance variables but central determinants of whether the minority fraction is captured and whether it can expand under assay conditions [38].

Emerging single-cell and droplet-based phenotyping methods directly address these limitations by measuring distributions rather than averages. Single-cell viability assays and microfluidic droplet systems can enumerate growth responses of very large numbers of individual cells, enabling sensitive detection of rare resistant subpopulations and explicit quantification of susceptibility distributions [38,55]. These approaches reveal clonal heterogeneity that is invisible to bulk AST and provide a conceptual blueprint for future diagnostics, although substantial engineering, standardization, and validation challenges remain before routine clinical deployment [4]. Importantly, many advanced diagnostic approaches discussed here, including PAP-AUC workflows, single-cell phenotyping, droplet microfluidics, and integrative omics-based pipelines, remain technically demanding, relatively low-throughput, expensive, and insufficiently standardized for routine implementation in many clinical microbiology laboratories.

A complementary strategy is kinetic phenotyping based on time-kill curves. Heteroresistance often manifests as biphasic killing, characterized by rapid elimination of the susceptible majority followed by regrowth driven by resistant minorities [4,14] (Figure 2). Semimechanistic and birth-death modeling of time-kill data can infer minority fractions, growth rates, and exposure thresholds that permit regrowth, parameters not captured by static MIC measurements [14,56]. Such models align with pharmacodynamic frameworks, including the mutant selection window concept [57], and offer interpretable links between *in vitro* dynamics and clinically achieved drug exposures [14].

Genomic and other omics approaches add mechanistic insight but face inherent constraints. Bulk whole-genome sequencing can miss low-frequency variants or unstable copy-number amplifications that drive heteroresistance, contributing to genotype–phenotype discordance near clinical breakpoints [2,58]. Genomic and within-host evolution studies that track copy number show that amplification-driven heteroresistance can arise and disappear dynamically in response to changing selective pressures [20]. Transcriptomic and proteomic analyses further show that inducible, reversible resistance-like states can generate heteroresistance without stable genetic change, suggesting that state-based biomarkers may sometimes be more informative than gene presence alone [6,8,16,59].

Artificial intelligence and machine-learning approaches increasingly serve as an integration layer across heterogeneous data modalities. However, current applications remain largely exploratory and require further validation across diverse pathogens, laboratories, and clinical settings before routine implementation. Models trained on genomic features and derived laboratory signals have shown improved ability to detect heteroresistance-associated patterns compared with rule-based gene detection alone [47]. In the near term, the most practical application is risk-based triage that assigns isolates a heteroresistance suspicion score using signals such as replicate MIC variability, early kinetic growth patterns, MALDI-TOF features, and genomic context [47,60]. High-risk isolates can then be routed to distribution-resolving confirmatory assays, while low-risk isolates follow standard workflows [4].

Taken together, current evidence supports a layered, pragmatic diagnostic strategy. Routine AST remains the first line for standardized susceptibility categorization, but its limitations must be recognized [4,5]. Targeted triage, followed by confirmatory distribution- or kinetics-based testing and mechanism-informed interpretation, offers a realistic path toward routine recognition of heteroresistance [4]. Fundamentally, because heteroresistance is dynamic and distributional, effective diagnostics must either directly measure population heterogeneity or reliably infer it from time-resolved and multimodal data, while minimizing pre-analytic steps that erase the very minority subpopulations that drive clinical risk [2,3].

## Heteroresistance as a transitional stage to stable resistance

A recurring clinical and evolutionary concern is that heteroresistance can function as a transitional stage that facilitates the emergence of stable resistance. Syntheses of animal infection studies indicate that resistant subpopulations, even when present at very low frequencies, can precipitate treatment failure, underscoring that numerically minor fractions may have disproportionate clinical impact when antibiotic exposure selectively enriches them. Importantly, heteroresistant populations can pre-exist before drug exposure and subsequently be selected during therapy, and evidence from clinical isolate studies supports that heteroresistance can precede and be supplanted by stable resistance over time, linking heteroresistance to evolutionary trajectories observed during treatment [13,37,48]. In a murine infection model, carbapenem-resistant *Klebsiella pneumoniae* with clinically undetected colistin heteroresistance caused treatment failure, demonstrating that heteroresistance can be both diagnostically cryptic and biologically decisive under antibiotic pressure [12]. Similarly, antibiotic failure mediated by resistant subpopulations has been demonstrated in *Enterobacter cloacae*, indicating that minority-driven treatment failure is not restricted to a single pathogen or drug class [11].

Conceptual and empirical work support the view that heteroresistance can act as an evolutionary intermediate. In particular, gene duplication/amplification can generate low-frequency, higher-resistance subpopulations rapidly and reversibly, providing a short-term adaptive benefit under antibiotic selection even though amplifications are often costly and unstable. Continued selection can then favor secondary sequence mutations that raise resistance or compensate costs so that high resistance becomes less dependent on the amplification state, and/or trajectories in which genetically stable resistance mutations emerge and eventually dominate, while the amplification can be reduced or lost. This provides a plausible mechanistic pathway by which a transient, dosage-based phenotype can transition into stable, high-level resistance during prolonged or repeated drug exposure [34,35,61,62].

## Conclusions

Heteroresistance is a population phenotype in which an isolate that appears largely susceptible contains rare subpopulations capable of growth at substantially higher antibiotic concentrations, creating a credible risk of treatment failure even when routine antimicrobial susceptibility testing classifies the isolate as susceptible. Evidence from reviews and quantitative studies indicates that heteroresistance is widespread across pathogens and drug classes and that its defining instability, expansion under antibiotic exposure, and contraction once selection is removed, makes it simultaneously clinically consequential and diagnostically elusive [1,3,4,37]. Mechanistically, heteroresistance spans a phenotypic-genotypic continuum. In many Gram-negative bacteria, genotypic heteroresistance is frequently driven by gene dosage variation (tandem gene amplification and plasmid copy-number shifts), whereas heteroresistance in *S. aureus* can arise from a broad set of point mutations in core genes [1,9]. Phenotypic heteroresistance reflects reversible physiological and regulatory state changes, with growing evidence for intergenerational ‘phenotypic memory’ of resistant states and for rapid transcriptional adaptation producing heteroresistance-like growth under β-lactams and related stresses [8,10]. Detection, therefore, remains a central translational bottleneck: emerging copy-number–aware genomics and integrative machine-learning approaches are promising for triage and surveillance, but they require rigorous external validation and careful clinical integration rather than stand-alone decision-making [47,58]. At present, standardized clinical interpretation frameworks for heteroresistance remain underdeveloped, and there is insufficient evidence to support universally accepted therapeutic algorithms based solely on heteroresistance detection. Finally, multiple lines of evidence support the view that heteroresistance can act as a transient stepping stone toward stable resistance under sustained or repeated selection by maintaining survival and population size long enough for compensatory change, additional mutations, or stabilization of previously unstable genetic states [7,63]. Future progress in understanding and managing heteroresistance will require tighter integration of evolutionary biology, clinical microbiology, and translational diagnostics.

Although the present review primarily focuses on bacterial heteroresistance, related forms of population-level antimicrobial survival have increasingly been recognized in fungal pathogens, providing an opportunity for broader comparative perspectives on antimicrobial population heterogeneity. Importantly, emerging work in fungal pathogens indicates that analogous population phenomena, termed antifungal persistence, also contribute to treatment failure despite apparent *in vitro* susceptibility. In *Candida*, *Aspergillus*, and other medically important fungi, small subpopulations can survive otherwise fungicidal drug exposure without stable resistance mutations, driven by stress-response rewiring, metabolic remodeling, biofilm-associated states, and epigenetic or chromatin-level regulation. The conceptual parallel between bacterial heteroresistance and fungal persistence underscores that population heterogeneity under antimicrobial stress is a cross-kingdom phenomenon with shared evolutionary logic but lineage-specific molecular underpinnings [64].

Conceptually, a key priority is to resolve how different forms of heteroresistance, genotypic, phenotypic, and hybrid, interact across timescales and treatment regimens and under what conditions they most strongly predispose populations to stable resistance. Quantitative frameworks that explicitly model population structure, gene dosage dynamics, phenotypic memory, and antibiotic pharmacodynamics will be essential to predict when heteroresistance meaningfully alters clinical outcomes rather than remaining an epiphenomenon. From a diagnostic perspective, the challenge is not merely technical sensitivity but clinical relevance. Next-generation sequencing approaches capable of detecting low-frequency variants and copy-number variation, alongside functional assays and machine-learning-based classifiers, must be benchmarked against patient outcomes and treatment responses. This will likely necessitate redefining susceptibility categories to incorporate population heterogeneity and evolutionary potential, rather than relying solely on binary growth thresholds.

Finally, heteroresistance has important implications for antimicrobial stewardship and drug development. If heteroresistant subpopulations routinely act as evolutionary reservoirs, then treatment strategies that suppress their expansion or exploit their instability may slow resistance emergence. Rational combination therapies, optimized dosing strategies, and drugs that target amplification or regulatory plasticity represent promising but underexplored avenues. Extending this logic to antifungal therapy, strategies aimed at preventing or disrupting persistent fungal subpopulations, such as targeting stress-response pathways, biofilm resilience mechanisms, or epigenetic regulators, may similarly reduce relapse and delay resistance evolution. Framing heteroresistance not as an anomaly but as a common, dynamic state of bacterial populations may ultimately shift both how resistance is monitored and how antibiotics are deployed in clinical practice.

## Abbreviations

AST: antimicrobial susceptibility testing
hVISA: heterogeneous vancomycin-intermediate *S. aureus*
MIC: minimum inhibitory concentration
PAP: population analysis profiling
PAP-AUC: population analysis profile-area under the curve
VISA: vancomycin-intermediate *S. aureus*
